# Metal accumulation in relation to size and body condition in an all-alien species community

**DOI:** 10.1007/s11356-021-17621-0

**Published:** 2021-12-01

**Authors:** Paride Balzani, Antonín Kouba, Elena Tricarico, Melina Kourantidou, Phillip J. Haubrock

**Affiliations:** 1 Department of Biology, University of Florence, Via Madonna del Piano 6, 50019 Sesto Fiorentino, Italy; 2 South Bohemian Research Center of Aquaculture and Biodiversity of Hydrocenoses, Faculty of Fisheries and Protection of Waters, University of South Bohemia in České Budějovice, Zátiší 728/II, 389 25 Vodňany, Czech Republic; 3 Hellenic Center for Marine Research, Institute of Marine Biological Resources and Inland Waters, 164 52 Athens, Greece; 4 Department of Sociology, Environmental and Business Economics, University of Southern Denmark, Degnevej 14, 6705 Esbjerg Ø, Denmark; 5 Department of River Ecology and Conservation, Senckenberg Research Institute and Natural History Museum Frankfurt, Clamecystrasse 12, 63571 Gelnhausen, Germany

**Keywords:** Environmental pollution, Bioaccumulation, Fish, Ecotoxicology, Freshwater ecosystems, Metals, Fulton condition factor

## Abstract

Metal pollution is one of the main environmental threats in freshwater ecosystems. Aquatic animals can accumulate these substances and transfer them across the food web, posing risks for both predators and humans. Accumulation patterns strongly vary depending on the location, species, and size (which in fish and crayfish is related to age) of individuals. Moreover, high metal concentrations can negatively affect animals’ health. To assess the intraspecific relationship between metal accumulation and size and health (proxied by the body condition) of individuals, the concentration of 14 metals (Al, As, Cd, Co, Cr, Cu, Fe, Hg, Mg, Mn, Ni, Pb, Se, Zn) was analyzed in six alien species from the highly anthropogenically altered Arno River (Central Italy): five fish (*Alburnus alburnus*, *Pseudorasbora parva*, *Lepomis gibbosus*, *Ictalurus punctatus*, and *Silurus glanis*) and one crayfish (*Procambarus clarkii*). We found that in *P. clarkii*, Cu was negatively related to size, as well as Al in *L. gibbosus* and Mg for adult *I. punctatus*. Positive size-dependent relationships were found for Hg in *L. gibbosus*, Fe in *S. glanis*, and Cr in juvenile *I. punctatus*. Only Co and Mg in *S. glanis* were found to negatively correlate with individual health. Since metal concentrations in animal tissue depend on trade-offs between uptake and excretion, the few significant results suggest different types of trade-offs across different species and age classes. However, only predatory fish species (*L. gibbosus*, *I. punctatus*, and *S. glanis*) presented significant relationships, suggesting that feeding habits are one of the primary drivers of metal accumulation.

**Supplementary Information:**

The online version contains supplementary material available at 10.1007/s11356-021-17621-0.

## Introduction

Metal and metalloid (hereafter “metal”) pollution is one of the most serious environmental hazards (Gall et al. 2015; Yang et al. 2018), posing both ecological and human health risks (Alhashemi et al. 2012; Liu et al. 2018, 2021). This threat originates from their uptake from the environment and subsequent bioaccumulation in animal tissues over time and their tendency to biomagnify through the transfer along the food chain to higher trophic positions (Markert et al. 2003; Madgett et al. 2021; Yang et al. 2021). While most metals are “essential” (i.e., needed for physiological functions as opposed to “non-essential” metals), high bioaccumulation causes detrimental effects on the health and fitness of aquatic animals (Reddy et al. 1997; Funes et al. 2006; Zeitoun and Mehana 2014; Javed and Usmani 2019), leading to behavioral, biochemical, and histological changes and potentially even death (Has-Schön et al. 2015; Fonseca et al. 2017; Greani et al. 2017).

Aquatic organisms accumulate metals from their local environment (i.e., water or sediment) either through their gills and skin, or through their digestive system after consumption of contaminated food sources (Squadrone et al. 2013; Has-Schön et al. 2015). Metal concentrations can be affected, among other factors, by the level of environmental contamination and the duration of exposure (Kouba et al. 2010; Has-Schön et al. 2015). Therefore, as organisms grow, it can be expected that larger (i.e., older) individuals have accumulated higher metal concentrations than smaller (i.e., younger) ones. Another factor potentially affecting metal bioaccumulation is the species richness and biomass of the recipient environment (McKinley and Johnston 2010); as prey-rich ecosystems typically have more diverse pathways, metals can more easily transfer along the food chain (Balzani et al. 2021). As such, it can be assumed that generalist predators relying on multiple prey species with diverse metal accumulation levels will express higher accumulation variability depending on the food web complexity, while at the same time differing from specialized consumers (Yevtushenko 1998).

The Arno River in Tuscany is the second biggest river in Central Italy. Particularly in Florence, the Arno River is anthropogenically and hydromorphologically altered (i.e., divided by weirs slowing its flow, channelization). The species community is characterized by a dominance of alien species, which have led to a complete species turnover from a native community to an almost all-alien species assemblage (Haubrock et al. 2021a). The fish assemblage, in particular, is entirely composed of alien species, some of which are considered invasive. Among these alien species, there are the two catfish species, *Silurus glanis* and *Ictalurus punctatus*, but also a variety of cyprinids (*Alburnus alburnus*, *Barbus barbus*, *Cyprinus carpio*, *Pseudorasbora parva*, *Tinca tinca*; *Squalius cephalus*) and others (*Lepomis gibbosus*, *Padogobius* sp.) (Haubrock et al. 2019a). Among the crustaceans, the invasive *Procambarus clarkii* and *Dikerogammarus villosus* are the most prominent in terms of abundance (Haubrock et al. 2019a). The river is contaminated from a variety of substances, comprising drugs (Zuccato et al. 2008), pesticides (Griffini et al. 1997), and metals (Cortecci et al. 2009) from both natural (i.e., weathering of metal-bearing rocks) and anthropogenic sources (i.e., industrial and agricultural activities) (Dinelli et al. 2005). In addition to those, nitrate derived from fertilizers, soil-organic, and wastewater origin have also been recorded (Nisi et al. 2005).

Thus far, little is known on how metals accumulate within alien species assemblages and how this may be affected by intraspecific characteristics (Balzani et al. 2021). Since alien species can generally tolerate higher pollutant concentrations than native species (El Haj et al. 2019), studying the sublethal effects on their fitness is an interesting avenue of research. To explore these relationships, we investigated intraspecific relationships for metal bioaccumulation in five fish and one crustacean species among the species present in the Arno River, using the alien species assemblage from this river as a model. We hypothesized that, in each species, larger and thus older individuals will have accumulated higher metal concentrations and that metal concentration will negatively correlate with body condition.

## Materials and methods

### Study site and sampling

**Fig. 1:**
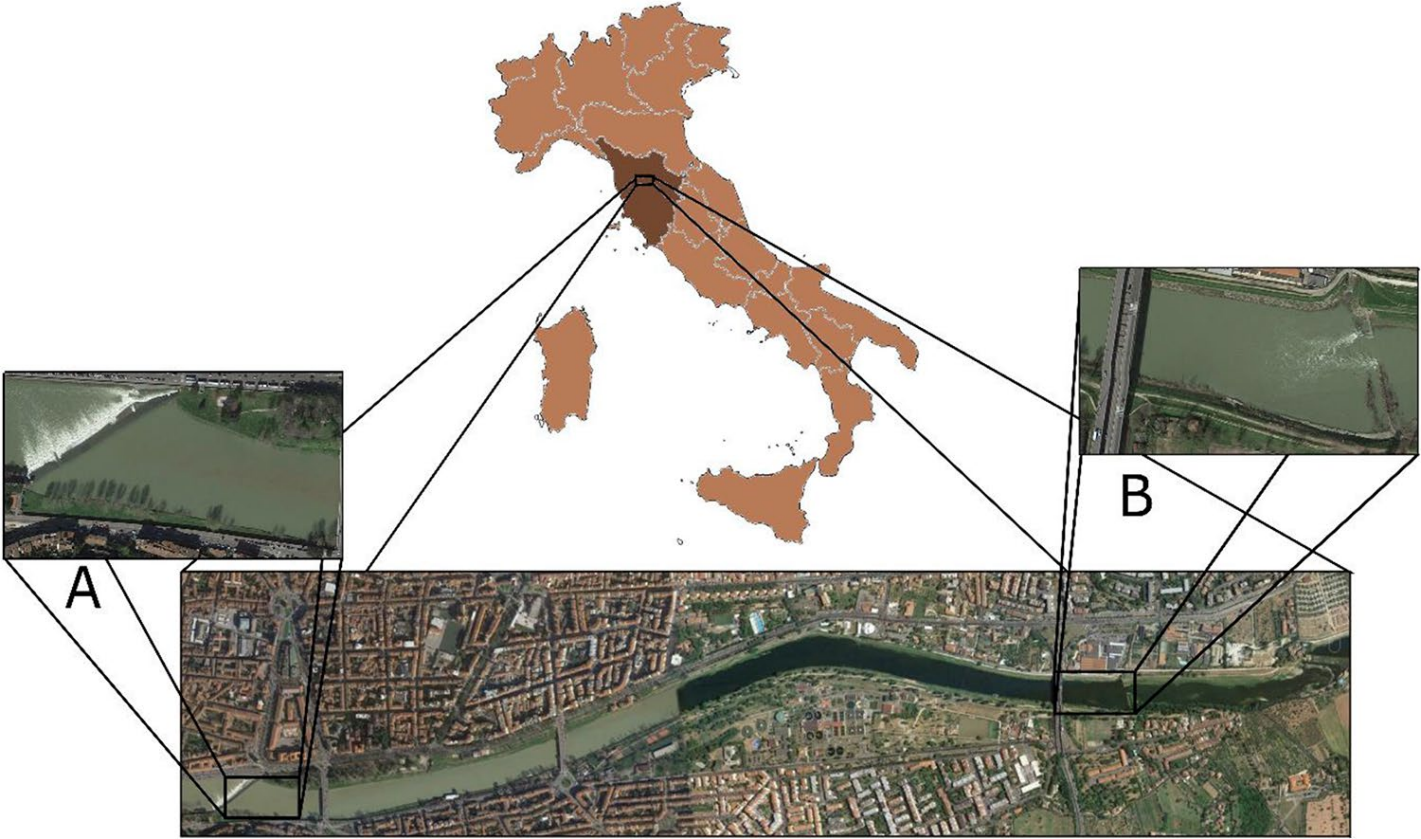
Map of the study site, which is a ~ 2.4 km stretch of the Arno River, showing the two weirs (A and B) enclosing the sampling area

The sampling was conducted from April to June 2018 within one stretch of the inner-Florence section of the Arno River (43.765606 N 11.268234 E, ~ 2.4 km length), which is delimitated by weirs (Fig. 1). Fish were caught with standard fishing rods using a variety of baits, and crayfish were caught using funnel traps. Caught fish were immediately euthanized via stunning, followed by gill cutting with a clean ceramic blade, while crayfish were killed by freezing, in compliance with the authorization (“Autorizzazione alla pesca scientifica Regione Toscana”). Samples were stored in ice during transport and then preserved in the freezer at − 20℃ until further processing. Overall, 110 individuals belonging to five fish and one crustacean alien species were collected: 7 *A. alburnus*, 4 *P. parva*, 16 *L. gibbosus*, 37 *S. glanis*, 36 *I. punctatus*, and 10 *P. clarkii*. Life stages of *I. punctatus* were distinguished following Haubrock et al. (2018), with specimens of a total length > 30 cm considered adults, resulting in a total of 16 juveniles and 20 adults caught. Only adults were caught for the other fish species based on primary and secondary sex characteristics.

For each individual crayfish, we measured the cephalothorax length (CTL; from the tip of the rostrum to the end of carapace, cm ± 0.1), while for each individual fish, we measured weight (W; g ± 0.1) and total length (TL; from the tip of the snout to the tip of the longer lobe of the caudal fin, cm ± 0.1) and calculated the Fulton factor (*K*), defined as *K* = 100*W/TL^3^. The Fulton factor is a morphometric index of body condition, commonly used as a proxy to assess the health and fitness condition of an individual in relation to the size of the species population (Froese 2006; Nash et al. 2006). Indeed, it is based on the fact that greater body mass at a given length corresponds to better conditions (Schloesser and Fabrizio 2017).

### Metal sample preparation and analysis

For the analysis of metals, a sample of abdominal muscle (for crayfish) and dorsal muscle without skin (for fish) was taken from each specimen. Samples were weighed wet, dried in an oven at 60℃ for 48 h, and weighed again dry. Organic matrices were prepared, weighing 50–500 mg (dry weight). Each sample was diluted in 10 ml of nitric acid, then mineralized with microwave radiation (1600 W, 210℃; Olesik 1991; Low et al. 2009; Ghanthimathi et al. 2012) to homogenize them and subsequently analyzed through Inductively Coupled Plasma – Optical Emission Spectrometry (ICP-OES). A total of 11 blanks (one every approximately 10 samples) were also prepared to control for contamination. Before running the analyses and at the end of each measurement session, certified standards of known metal concentrations (multistandard concentrations: 0.1 ppm, 1 ppm, and 10 ppm; Hg standard concentrations: 0.01 ppm and 0.05 ppm) were used to calibrate the instruments and to ensure that no instrumental bias occurred. In compliance with quality assurance and quality control (QA/QC), three replicates for each sample (from the same digestion solution) were run and their relative standard deviations (RSD) were calculated. The respective mean metal concentration was used for further analyses.

For each sample, the following metal concentrations were determined: aluminum (Al), arsenic (As), cadmium (Cd), cobalt (Co), chromium (Cr), copper (Cu), iron (Fe), mercury (Hg), magnesium (Mg), manganese (Mn), nickel (Ni), lead (Pb), selenium (Se), and zinc (Zn). The analytical detection limit for all metals was 0.01 ppm on a dry weight basis. Concentrations in blanks were < 1% of the samples, and all the RSDs were < 10%.

### Statistical analyses

Before running statistical analyses, each value of samples that presented metal concentrations below the detection limit (0.01) was substituted with the value of the detection limit itself (Soto et al. 2016) and metal concentrations were log_10_-transformed to account for multiplicative effects.

To display correlations, an explorative correlation analysis using Spearman’s rank correlation was performed (R package “corrplot”; Wei et al. 2017) for each species. A preliminary linear model on log_10_-transformed total length and weight was performed for fish. Since a significant relationship was found (*F*_1,197_ = 2460.4, *p* < 0.001, adj. *R*^2^ = 0.93), only total length was used for subsequent analyses. To identify relationships between metal concentrations, size, and body condition, we built a linear model for each species using the “step” function for every log_10_-transformed metal concentration as response variable and length (TL or CTL) and Fulton factor (K) as predictors. All statistical analyses were performed using the software R (4.0 version, R Core Team 2020), and the level of statistical significance (α) was set at *p* = 0.05.

## Results

**Fig. 2:**
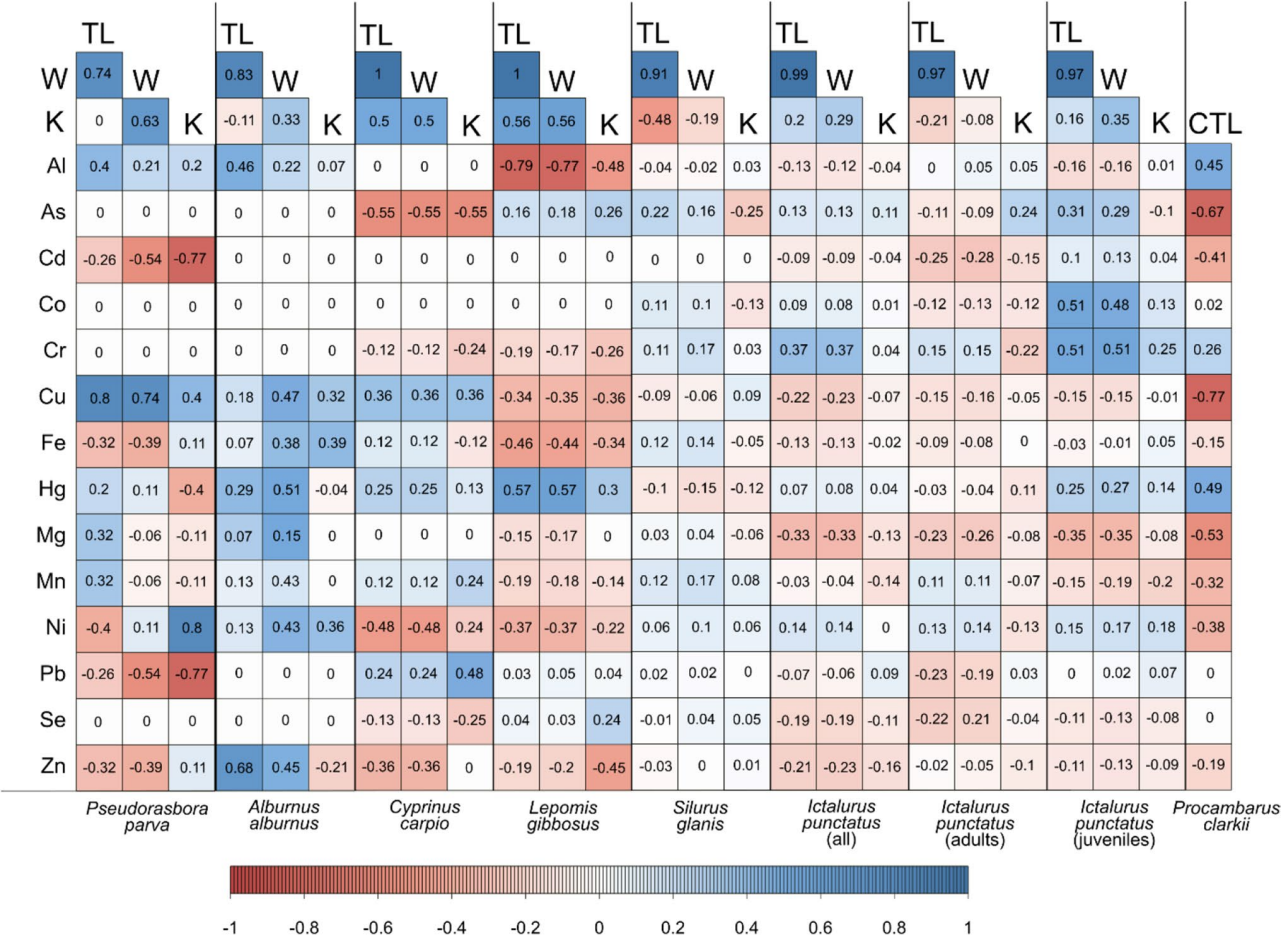
Correlation plot of all metal concentrations with length (CTL, cephalothorax length for crayfish; TL, total length for fish), weight (W), and Fulton factor (K) for each species

All the correlations of the measured metals with length, weight, and Fulton factor for every species are shown in Fig. 2.

The applied models showed significant relationships for only four species (*P. clarkii*, *L. gibbosus*, *S. glanis*, and *I. punctatus*). The cyprinid species *A. alburnus* and *P. parva* did not show a significant relationship with any metal (Supplementary information 1). Only a few metals were found to significantly correlate with length (Cu for *P. clarkii*, Al and Hg for *L. gibbosus*, Fe for *S. glanis*, and Cr and Mg for juveniles and adults, respectively, of *I. punctatus*). Only two metals (Co and Mg) in one species (*S. glanis*) were found to significantly affect the species’ health as proxied by the Fulton factor (Table 1). While the relationships with the Fulton factor were both negative, the relationships with length were, depending on the metal, both positive (for Hg in *L. gibbosus*, Fe in *S. glanis*, and Cr in juvenile *I. punctatus*) and negative (for Cu in *P. clarkii*, Al in *L. gibbosus*, Mg in adult *I. punctatus*).

**Table 1:** Results of the significant linear models with log_10_-transformed metal concentration as response variable and length (CTL, cephalothorax length for crayfish; TL, total length for fish) and Fulton factor (K) as predictors

Species	Metal	Covariate	Estimate	Standard error	*t*-value	*p*	*F*	Adj. *R*^2^
*Procambarus clarkii*	Cu	Intercept	2.39	0.16	15.12	< 0.001***	*F*_1,8_ = 15.89	0.62
		CTL	− 0.09	0.02	− 3.99	0.004**		
*Lepomis gibbosus*	Al	Intercept	2.17	0.29	7.55	< 0.001***	*F*_1,14_ = 20.55	0.57
		TL	− 0.14	0.03	− 4.53	< 0.001***		
	Hg	Intercept	− 3.24	0.78	− 4.17	< 0.001***	*F*_1,14_ = 9.25	0.35
		TL	0.25	0.08	3.04	< 0.01**		
*Silurus glanis*	Co	Intercept	− 1.27	0.26	− 4.88	< 0.001***	*F*_1,35_ = 6.46	0.13
		K	− 1.18	0.46	− 2.54	0.02*		
	Fe	Intercept	0.92	0.17	5.38	< 0.001***	*F*_1,35_ = 4.14	0.08
		TL	0.01	0.01	2.04	0.049*		
	Mg	Intercept	3.53	0.20	17.74	< 0.001***	*F*_2,34_ = 3.94	0.14
		TL	− 0.01	0.004	− 2.44	0.11		
		K	− 0.50	0.22	− 2.29	0.03*		
*Ictalurus punctatus* (juveniles and adults)	Cr	Intercept	− 1.88	0.31	− 6.13	< 0.001***	*F*_1,34_ = 13.57	0.26
		TL	0.03	0.01	3.68	< 0.001***		
*Ictalurus punctatus* (juveniles)	Cr	Intercept	− 3.92	0.39	− 10.14	< 0.001***	*F*_1,14_ = 51.79	0.77
		TL	0.11	0.02	7.20	< 0.001***		
*Ictalurus punctatus* (adults)	Mg	Intercept	3.06	0.09	34.44	< 0.001***	*F*_1,18_ = 10.91	0.34
		TL	− 0.01	0.002	− 3.30	< 0.01**		

Asterisks refer to the significance level: *p* < 0.05 (*); *p* < 0.01 (**); *p* < 0.001 (***)

## Discussion

There is evidence that some metals bioaccumulate within organisms through time, leading to positive size and age-dependent relationships (Dragun et al. 2007; Rajkowska and Protasowicki 2013; Has-Schön et al. 2015). Among these, Hg, one of the most toxic metals even at low concentrations (Kaus et al. 2017; Waheed et al. 2020), is the one that most frequently follows this behavior (Squadrone et al. 2013; Zrnčić et al. 2013; Donadt et al. 2021). However, these relationships are not always obvious. In line with other studies (Jovičić et al. 2015; Léopold et al. 2015; Jia et al. 2017), we found only a few significant relationships between metal concentrations and total length (a proxy for age). In line with previous literature (Rakocevic et al. 2018), significant relationships were mainly found for essential elements, while no clear or not-significant relationships were found for the non-essentials. The absence of clear patterns is likely due to fish belonging to the same age class (adults) except for *I. punctatus*, for which comparable concentrations in both young and old animals were found. This latter result could be due to different reasons: the young for higher metabolism and ingestion rate, the old for the longer exposure to pollutants (Yi and Zhang 2012; Liu et al. 2015; Jia et al. 2017), and for predators, the greater consumption of contaminated prey from higher trophic levels (Balzani et al. 2021). Moreover, the variability we identified in the sign of metal–size relationships is also in line with other studies (Jezierska and Witeska 2001; Dragun et al. 2016; Jia et al. 2017). Even the same species can show different accumulation patterns for the same metal depending on the location or season (Barak and Mason 1990; Farkas et al. 2003; Noël et al. 2013; Ghosn et al. 2020).

Negative relationships are generally more frequent for alkaline elements (e.g., Li, Na, and K), whereas positive relationships are more commonly found for transition elements (e.g., Mn, Fe, and Co; Dragun et al. 2016; Jiang et al. 2022). Indeed, we found a negative relationship with the length for Cu (in *P. clarkii*) and Mg (in *I. punctatus* and *S. glanis*) and positive for Fe (in *S. glanis*), Hg (in *L. gibbosus*), and Cr (in *I. punctatus*). Partially in line with previous studies (Merciai et al. 2014; Jiang et al. 2022), we found negative relationships with the length for two essential metals (Cu in *P. clarkii* and Mg in *I. punctatus* and *S. glanis*) and positive relationships for two essential (Fe in *S. glanis* and Cr in *I. punctatus*) and one non-essential metal (Hg in *L. gibbosus*). Negative relationships could be indicative of faster metabolism in younger fish compared to older ones (Léopold et al. 2015) and higher tissue growing rate than metal uptake rate (Merciai et al. 2014; Dragun et al. 2016). In addition to that, a better metal bioregulation could occur in older fish (De Wet et al. 1994; Merciai et al. 2014). On the other hand, positive relationships can be due to a constant uptake and slow excretion rates (Has-Schön et al. 2015) as metal bioaccumulation depends on a trade-off between uptake and excretion (Adams et al. 2020), suggesting that our findings are likely the outcome of different trade-offs.

Crayfish size is known to correlate with some metal concentrations, especially Hg (Kouba et al. 2010). However, in this study, we found a significant (negative) relationship only for Cu. Although high Cu concentrations were found to be detrimental in *P. clarkii* (Bini and Chelazzi 2006; Zhao et al. 2019a), muscle has a slow Cu uptake rate compared to other tissues (Soedarini et al. 2012) and Cu elimination seems to be quite efficient in this species (Zhao et al. 2019b), resulting in low accumulation after exposure (Maranhão et al. 1995).

High metal concentrations within an organism can negatively affect its health (Wu et al. 2016; Fonseca et al. 2017), which can be proxied by the Fulton factor, that is used to assess body conditions (Froese 2006; Mozsár et al. 2015). However, in line with other studies (Jovičić et al. 2015), we found that the Fulton factor was mostly unaffected by metal concentration. The only exceptions were Mg and Co in *S. glanis*, which showed negative relationships, suggesting that life-history changes may play a considerable role. Previous literature showed, however, that this relationship can be highly variable (Alhashemi et al. 2012; Luczynska et al. 2016; Dragun et al. 2016; Rakocevic et al. 2018). One possible explanation is that fish could have physiological mechanisms that reduce the impact of metals on body condition (Tenji et al. 2020). It should also be noted that the Fulton factor is correlated with the body fat content (Schloesser and Fabrizio 2017), which can vary between individuals and species, and that metal accumulation can be positively or negatively related to the lipid content, depending on the metal (Sassd 2011; Charette et al. 2021).

Interspecific (as well as intraspecific, among tissues) differences in metal accumulation compared with the environmental concentrations and biomagnification processes in the Arno River community were disentangled in a recent study by Balzani et al. (2021). Nonetheless, by comparing intraspecific relationships, additional information on interspecific patterns can be derived. Interestingly, besides the omnivorous crayfish *P. clarkii*, only predatory fish species presented some significant relationships, whereas the more opportunistic cyprinids (*A. alburnus* and *P. parva*) did not. Considered separately, the two age classes of *I. punctatus* revealed additional information. First, the variance explained for the relationship between Cr and total length is much higher. Second, the relationship between Mg and total length was found to be significant for adult specimens. These observations could be due to ontogenetic differences in habitat use, behavior, and diet (Haubrock et al. 2018, 2020, 2021b) that are reflected in different metal accumulations (Balzani et al. 2021). Indeed, *I. punctatus* juveniles live close to the riverbank and feed more on detritus, while adults live on the bottom and feed in the whole water column (Endo et al. 2015; Haubrock et al. 2020), possibly leading to Cr bioaccumulation in young individuals and a reduction with age in Mg concentrations in adults. However, *S. glanis*, which similarly to adults of *I. punctatus* occupies benthic habitats but expresses pelagic feeding (Haubrock et al. 2020; De Santis and Volta 2021), showed higher Fe bioaccumulation with increasing age. Also, the riparian species *L. gibbosus* and *P. clarkii* (Donato et al. 2018; Bissattini et al. 2021; Haubrock et al. 2021c) showed negative relationships between metal concentrations and size. Therefore, the role of the living or feeding habitat in bioaccumulation is not straightforward and seems to vary according to the involved species.

Most of the alien species examined in this study have not been subject to management in the Arno River, except for *S. glanis*, a popular fish among anglers who practice “catch-and-release,” which used to be managed in the past (Arlinghaus et al. 2007; Cerri et al. 2018). Additionally, although none of those species is officially known to be harvested for commercial purposes or for human consumption in smaller quantities, anecdotal evidence suggests that the latter may be true (see also Squadrone et al. 2013). The lack of understanding of metal accumulation and interactions with size and body condition or health of these invasive species, together with the lack of management, therefore, poses a simultaneous risk to human health and to the already stressed ecosystem. The results of our study, in combination with earlier works shedding light on trophic interactions (Haubrock et al. 2019a), bioaccumulation and mechanisms through which those metals transfer across food webs to higher trophic levels (Balzani et al. 2021), can provide valuable input not only for conservation authorities concerned with impact mitigation but also for public health and food safety authorities. Indeed, despite the fact that several metal concentrations were found to be below the maximum permitted levels for human consumption as determined by the European Commission (Balzani et al. 2021; European Commission 2008), for metals such as Hg, there is evidence for considerable bioaccumulation across the trophic chain (Balzani et al. 2021). Mercury was also found in this study to have a significant positive relationship with the length of *L. gibbosus* which may indicate for example that any attempts to control the invasion, e.g., through removal/harvesting or other, could be targeted at earlier life stages. This result may also factor into management considerations for *P. clarkii* which is a key prey for *L. gibbosus* (Haubrock et al. 2019b) and for *I. punctatus* (adults), for which *L. gibbosus* is a key prey (Balzani et al. 2021).

At the same time, such works can help build a baseline for understanding interactions between these species and metals in other places where invasive populations have been established. One of the examined species for which significant relationships were identified (*P. clarkii*), owing to its high impacts and costs (Haubrock et al. 2021d; Kourantidou et al. 2021), is listed among the worst invasive species in Europe (Nentwig et al. 2018) and in the Union list of invasive species of concern attached to the EU Regulation 1143/2014 on invasive alien species (the list of invasive species for which management actions are mandatory). With almost no native fish species left in the Arno River (Balzani et al. 2020; Haubrock et al. 2021a), our finding that predatory fish species were the only ones with significant relationships, suggesting that feeding habitats are likely among the primary drivers of metal accumulation, is key to future restoration initiatives. Indeed, despite the poor environmental quality status of the Arno River, several ecosystem services, which include supporting services related to aquatic biodiversity in the river and its main tributaries, seem to be of great importance to nearby communities (Pacetti et al. 2020), reinforcing the need for restoration actions that require an adequate understanding of the underlying ecological mechanisms.

Last, it should be acknowledged that, in our study, the paucity of significant relationships could be the result of the small sample number, or the too narrow range of sizes sampled, thus representing only one age class. However, the importance of our contribution lies in the use of linear models that help ensure only robust relationships, as opposed to correlation analysis typically used to test such relationships, which can lead to overestimating the significant relationships. Nevertheless, increasing knowledge of patterns of metal bioaccumulation represents an important contribution to the environmental monitoring of freshwater ecosystems. Our work points at the need for more studies comparing native and alien populations to help identify stressors that contribute to underlying processes.

## Supplementary Information

Below is the link to the electronic supplementary material.

Supplementary file1 (DOCX 45 kb)

## Data Availability

The datasets used and/or analyzed during the current study are available from the corresponding author on reasonable request.
