# Supplementary material for: Metal accumulation in relation to size and body condition in an all-alien species community
Source: Environ Sci Pollut Res Int. 2021 Dec 1;29(17):25848–57. doi: 10.1007/s11356-021-17621-0 (PMC8986740; doi:10.1007/s11356-021-17621-0)
Supplement: Supplementary file 1 — Supplementary file1 (DOCX 45 kb) [file 11356_2021_17621_MOESM1_ESM.docx]

**Supplement 1. Summary of the modelling outputs (built using the ‘step’ function) for each species, for each metal concentration (after log10 transformation) using the total length and Fulton condition factor as predictors.**

**Alburnus alburnus**

| **metal** | **covariate** | **estimate** | **Standard error** | **t-value** | **p** | **F** | **Adj. R^2** |
| --- | --- | --- | --- | --- | --- | --- | --- |
| Al | Intercept | 0.49 | 0.34 | 1.46 | 0.19 | - | - |
| As | NA | NA | NA | NA | NA | NA | NA |
| Cd | NA | NA | NA | NA | NA | NA | NA |
| Co | NA | NA | NA | NA | NA | NA | NA |
| Cr | NA | NA | NA | NA | NA | NA | NA |
| Cu | Intercept | 0.42 | 0.14 | 3.07 | 0.02 * | - | - |
| Fe | Intercept | 1.14 | 0.56 | 2.05 | 0.09 | - | - |
| Hg | Intercept | -1.17 | 0.41 | -2.87 | 0.03 * | - | - |
| Mg | Intercept | 2.11 | 0.70 | 3.03 | 0.02 * | - | - |
| Mn | Intercept | 0.14 | 0.40 | 0.35 | 0.74 | - | - |
| Ni | Intercept | -0.95 | 0.34 | -2.83 | 0.03 | - | - |
| Pb | NA | NA | NA | NA | NA | NA | NA |
| Se | NA | NA | NA | NA | NA | NA | NA |
| Zn | Intercept | -0.33 | 1.29 | -0.26 | 0.81 | F_1,5_ = 2.97 | 0.25 |
|  | Total length | 0.23 | 0.13 | 1.72 | 0.15 |  |  |

**Cyprinus carpio**

| **metal** | **covariate** | **estimate** | **Standard error** | **t-value** | **p** | **F** | **Adj. R^2** |
| --- | --- | --- | --- | --- | --- | --- | --- |
| Al | NA | NA | NA | NA | NA | NA | NA |
| As | NA | NA | NA | NA | NA | NA | NA |
| Cd | NA | NA | NA | NA | NA | NA | NA |
| Co | NA | NA | NA | NA | NA | NA | NA |
| Cr | NA | NA | NA | NA | NA | NA | NA |
| Cu | NA | NA | NA | NA | NA | NA | NA |
| Fe | NA | NA | NA | NA | NA | NA | NA |
| Hg | NA | NA | NA | NA | NA | NA | NA |
| Mg | NA | NA | NA | NA | NA | NA | NA |
| Mn | NA | NA | NA | NA | NA | NA | NA |
| Ni | NA | NA | NA | NA | NA | NA | NA |
| Pb | NA | NA | NA | NA | NA | NA | NA |
| Se | NA | NA | NA | NA | NA | NA | NA |
| Zn | NA | NA | NA | NA | NA | NA | NA |

**Lepomis gibbosus**

| **metal** | **covariate** | **estimate** | **Standard error** | **t-value** | **p** | **F** | **Adj. R^2** |
| --- | --- | --- | --- | --- | --- | --- | --- |
| Al | Intercept | 2.17 | 0.29 | 7.55 | < 0.001 *** | F_1,14_ = 20.55 | 0.57 |
|  | Total length | -0.14 | 0.03 | -4.53 | < 0.001 *** |  |  |
| As | Intercept | -0.99 | 0.21 | -4.72 | < 0.001 *** | - | - |
| Cd | NA | NA | NA | NA | NA | NA | NA |
| Co | NA | NA | NA | NA | NA | NA | NA |
| Cr | Intercept | -1.19 | 0.43 | -2.78 | 0.01 * | F_1,14_ = 2.33 | 0.08 |
|  | Total length | -0.07 | 0.05 | -1.53 | 0.15 |  |  |
| Cu | Intercept | 0.14 | 0.22 | 0.65 | 0.53 | - | - |
| Fe | Intercept | 1.37 | 0.24 | 5.78 | < 0.001 *** | - | - |
| Hg | Intercept | -3.24 | 0.78 | -4.17 | < 0.001 *** | F_1,14_ = 9.25 | 0.35 |
|  | Total length | 0.25 | 0.08 | 3.04 | < 0.01 ** |  |  |
| Mg | Intercept | 2.33 | 0.42 | 5.51 | < 0.001 *** | - | - |
| Mn | Intercept | -0.16 | 0.20 | -0.81 | 0.43 | - | - |
| Ni | Intercept | -0.85 | 0.17 | -5.15 | < 0.001 *** | - | - |
| Pb | NA | -1.81 | 0.11 | -16.71 | < 0.001 *** | - | - |
| Se | NA | -0.83 | 0.31 | -2.71 | 0.02 * | - | - |
| Zn | Intercept | 1.19 | 0.31 | 3.81 | < 0.01 ** | - | - |

**Pseudorasbora parva**

| **metal** | **covariate** | **estimate** | **Standard error** | **t-value** | **p** | **F** | **Adj. R^2** |
| --- | --- | --- | --- | --- | --- | --- | --- |
| Al | Intercept | 0.82 | 0.17 | 4.81 | 0.02 * | - | - |
| As | NA | NA | NA | NA | NA | NA | NA |
| Cd | Intercept | -1.70 | 0.30 | -5.65 | 0.01 * | - | - |
| Co | NA | NA | NA | NA | NA | NA | NA |
| Cr | NA | NA | NA | NA | NA | NA | NA |
| Cu | Intercept | 0.50 | 0.24 | 2.07 | 0.13 | - | - |
| Fe | Intercept | -0.10 | 1.10 | -0.09 | 0.93 | - | - |
| Hg | Intercept | -1.13 | 0.34 | -3.34 | 0.04 * | - | - |
| Mg | Intercept | 0.47 | 1.43 | 0.33 | 0.76 | - | - |
| Mn | Intercept | -0.74 | 0.73 | -1.01 | 0.39 | - | - |
| Ni | Intercept | 3.81 | 2.49 | -1.53 | 0.27 | F_1,2_ = 1.45 | 0.13 |
|  | Fulton factor | 3.74 | 3.10 | 1.21 | 0.35 |  |  |
| Pb | Intercept | -1.42 | 0.58 | -2.46 | 0.09 | - | - |
| Se | NA | NA | NA | NA | NA | NA | NA |
| Zn | Intercept | 0.01 | 1.16 | 0.01 | 1 | - | - |

**Silurus glanis**

| **metal** | **covariate** | **estimate** | **Standard error** | **t-value** | **p** | **F** | **Adj. R^2** |
| --- | --- | --- | --- | --- | --- | --- | --- |
| Al | Intercept | 0.57 | 0.06 | 8.92 | < 0.001 *** | - | - |
| As | Intercept | -1-94 | 0.43 | -4.49 | < 0.001 *** | F_1,35_ = 2.04 | 0.03 |
|  | Total length | 0.02 | 0.02 | 1.43 | 0.16 |  |  |
| Cd | NA | NA | NA | NA | NA | NA | NA |
| Co | Intercept | -1.27 | 0.26 | -4.88 | < 0.001 *** | F_1,35_ = 6.46 | 0.13 |
|  | Fulton factor | -1.18 | 0.46 | -2.54 | 0.02 * |  |  |
| Cr | Intercept | -1.10 | 0.13 | -8.18 | < 0.001 *** | - | - |
| Cu | Intercept | 0.05 | 0.04 | 1.50 | 0.14 | - | - |
| Fe | Intercept | 0.92 | 0.17 | 5.38 | < 0.001 *** | F_1,35_ = 4.14 | 0.08 |
|  | Total length | 0.01 | 0.01 | 2.04 | 0.049 * |  |  |
| Hg | Intercept | 0.09 | 0.40 | 0.22 | 0.83 | F_1,35_ = 3.34 | 0.06 |
|  | Total length | -0-03 | 0.01 | -1.83 | 0.08 |  |  |
| Mg | Intercept | 3.53 | 0.20 | 17.74 | < 0.001 *** | F_2,34_ = 3.94 | 0.14 |
|  | Total length | -0.01 | 0.004 | -2.44 | 0.11 |  |  |
|  | Fulton factor | -0.50 | 0.22 | -2.29 | 0.03 * |  |  |
| Mn | Intercept | 0.14 | 0.07 | 1.85 | 0.07 | - | - |
| Ni | Intercept | -2.19 | 0.55 | -3.98 | < 0.001 *** | F_2,34_ = 3.62 | 0.13 |
|  | Fulton factor | 1.54 | 0.61 | 2.52 | 0.07 |  |  |
|  | Total length | 0.02 | 0.01 | 1.90 | 0.07 |  |  |
| Pb | Intercept | -2.21 | 0.20 | -11.23 | < 0.001 *** | F_1,35_ = 2.18 | 0.03 |
|  | Total length | 0.01 | 0.01 | 1.48 | 0.15 |  |  |
| Se | Intercept | -1.83 | 0.09 | -19.35 | < 0.001 *** | - | - |
| Zn | Intercept | 1.50 | 0.03 | 49.55 | < 0.001 *** | - | - |

**Ictalurus punctatus juveniles**

| **metal** | **covariate** | **estimate** | **Standard error** | **t-value** | **p** | **F** | **Adj. R^2** |
| --- | --- | --- | --- | --- | --- | --- | --- |
| Al | Intercept | 0.50 | 0.14 | 3.69 | < 0.01 ** | - | - |
| As | Intercept | -1.83 | 0.10 | -18.16 | < 0.001 *** | - | - |
| Cd | Intercept | -1.94 | 0.06 | -30.51 | < 0.001 *** | - | - |
| Co | Intercept | -1.95 | 0.04 | -52.54 | < 0.001 *** |  |  |
| Cr | Intercept | -3.92 | 0.39 | -10.14 | < 0.001 *** | F_1,14_ = 51.79 | 0.77 |
|  | Total length | 0.11 | 0.02 | 7.20 | < 0.001 *** |  |  |
| Cu | Intercept | 0.14 | 0.15 | 0.98 | 0.34 | - | - |
| Fe | Intercept | 1.23 | 0.22 | 5.60 | < 0.001 *** |  |  |
| Hg | Intercept | -1.10 | 0.56 | -1.97 | 0.07 | F_1,14_ = 2.39 | 0.08 |
|  | Total length | 0.04 | 0.02 | 1.55 | 0.14 |  |  |
| Mg | Intercept | 2.57 | 0.31 | 8.42 | < 0.001 *** | - | - |
| Mn | Intercept | -0.03 | 0.14 | -0.23 | 0.82 | - | - |
| Ni | Intercept | -0.76 | 0.11 | -7.07 | < 0.001 *** | - | - |
| Pb | Intercept | -1.65 | 0.13 | -12.34 | < 0.001 *** | - | - |
| Se | Intercept | -1.71 | 0.20 | -8.70 | < 0.001 *** | - | - |
| Zn | Intercept | 1.38 | 0.23 | 6.07 | < 0.001 *** | - | - |

**Ictalurus punctatus adults**

| **metal** | **covariate** | **estimate** | **Standard error** | **t-value** | **p** | **F** | **Adj. R^2** |
| --- | --- | --- | --- | --- | --- | --- | --- |
| Al | Intercept | 0.50 | 0.10 | 4.98 | < 0.001 *** | - | - |
| As | Intercept | -1.92 | 0.06 | -30.4 | < 0.001 *** |  |  |
| Cd | NA | NA | NA | NA | NA | NA | NA |
| Co | Intercept | -1.99 | 0.01 | -185.2 | < 0.001 *** | - | - |
| Cr | Intercept | -0.50 | 0.12 | -4.06 | < 0.001 *** | - | - |
| Cu | Intercept | 0.27 | 0.03 | 10.02 | < 0.001 *** | - | - |
| Fe | Intercept | 1.42 | 0.05 | 30.37 | < 0.001 *** | - | - |
| Hg | Intercept | 0.36 | 0.37 | 0.98 | 0.34 | F_1,18_ = 2.35 | 0.07 |
|  | Total length | -0.01 | 0.01 | -1.53 | 0.14 |  |  |
| Mg | Intercept | 3.06 | 0.09 | 34.44 | < 0.001 *** | F_1,18_ = 10.91 | 0.34 |
|  | Total length | -0.01 | 0.002 | -3.30 | < 0.01 ** |  |  |
| Mn | Intercept | -0.06 | 0.03 | -2.35 | 0.03 * | - | - |
| Ni | Intercept | -0.56 | 0.08 | -7.35 | < 0.001 *** | - | - |
| Pb | Intercept | -1.07 | 0.47 | -2.30 | 0.03 * | F_1,18_ = 2.26 | 0.06 |
|  | Total length | -0.02 | 0.01 | -1.50 | 0.15 |  |  |
| Se | NA | NA | NA | NA | NA | NA | NA |
| Zn | Intercept | 1.50 | 0.02 | 80.94 | < 0.001 *** | - | - |

**Ictalurus punctatus overall**

| **metal** | **covariate** | **estimate** | **Standard error** | **t-value** | **p** | **F** | **Adj. R^2** |
| --- | --- | --- | --- | --- | --- | --- | --- |
| Al | Intercept | 0.50 | 0.08 | 6.17 | < 0.001 *** | - | - |
| As | Intercept | -1.88 | 0.06 | -33.26 | < 0.001 *** | - | - |
| Cd | Intercept | -1.97 | 0.03 | -69.91 | < 0.001 *** | - | - |
| Co | Intercept | -1.97 | 0.02 | -112.5 | < 0.001 *** | - | - |
| Cr | Intercept | -1.88 | 0.31 | -6.13 | < 0.001 *** | F_1,34_ = 13.57 | 0.26 |
|  | Total length | 0.03 | 0.01 | 3.68 | < 0.001 *** |  |  |
| Cu | Intercept | 0.21 | 0.07 | 3.2 | < 0.01 ** | - | - |
| Fe | Intercept | 1.33 | 0.10 | 13.29 | < 0.001 *** | - | - |
| Hg | Intercept | 0.22 | 0.08 | -2.98 | < 0.01 ** | - | - |
| Mg | Intercept | 2.69 | 0.13 | 19.91 | < 0.001 *** | - | - |
| Mn | Intercept | -0.05 | 0.06 | -0.78 | 0.44 | - | - |
| Ni | Intercept | -0.65 | 0.07 | -9.95 | < 0.001 *** | - | - |
| Pb | Intercept | -1.71 | 0.08 | -21.68 | < 0.001 *** | - | - |
| Se | Intercept | -1.47 | 0.25 | -5.97 | < 0.001 *** | F_1,34_ = 3.03 | 0.05 |
|  | Total length | -0.01 | 0.01 | -1.74 | 0.09 |  |  |
| Zn | Intercept | 1.45 | 0.10 | 14.43 | < 0.001 *** | - | - |

**Procambarus clarkii**

| **metal** | **covariate** | **estimate** | **Standard error** | **t-value** | **p** | **F** | **Adj. R^2** |
| --- | --- | --- | --- | --- | --- | --- | --- |
| Al | Intercept | 1.51 | 0.43 | 3.54 | 0.008 ** | F_1,8_ = 2.24 | 0.12 |
|  | CTL | 0.09 | 0.06 | 1.50 | 0.17 |  |  |
| As | Intercept | 0.87 | 0.62 | 1.41 | 0.20 | F_1,8_ = 3.07 | 0.19 |
|  | CTL | -0.16 | 0.09 | -1.75 | 0.12 |  |  |
| Cd | Intercept | -2.02 | 0.02 | -86.63 | < 0.001 *** | - | - |
| Co | Intercept | -1.74 | 0.15 | -11.67 | < 0.001 *** | - | - |
| Cr | Intercept | -1.06 | 0.28 | -3.84 | 0.004 ** | - | - |
| Cu | Intercept | 2.39 | 0.16 | 15.12 | < 0.001 *** | F_1,8_ = 15.89 | 0.62 |
|  | CTL | -0.09 | 0.02 | -3.99 | 0.004 ** |  |  |
| Fe | Intercept | 2.39 | 0.13 | 17.7 | < 0.001 *** | - | - |
| Hg | Intercept | -1.61 | 0.20 | -7.96 | < 0.001 *** | - | - |
| Mg | Intercept | 3.57 | 0.19 | 18.46 | < 0.001 *** | F_1,8_ = 2.61 | 0.15 |
|  | CTL | -0.05 | 0.03 | -1.61 | 0.15 |  |  |
| Mn | Intercept | 1.15 | 0.17 | 6.97 | < 0.001 *** | - | - |
| Ni | Intercept | -0.15 | 0.26 | -0.58 | 0.57 | - | - |
| Pb | NA | NA | NA | NA | NA | NA | NA |
| Se | NA | NA | NA | NA | NA | NA | NA |
| Zn | Intercept | 1.46 | 0.39 | 3.80 | 0.004 ** | - | - |
